# A gross anatomic study of distal tibia and fibula for single-incision approach

**DOI:** 10.1186/1749-799X-9-28

**Published:** 2014-04-24

**Authors:** Hui Ma, Jie Zhao, Baoqing Yu, Bin Ye

**Affiliations:** 1 Department of Orthopaedic Surgery, Shanghai First Rehabilitation Hospital, No. 349 Hangzhou Road, Yangpu District, Shanghai 200090, People's Republic of China; 2 Department of Orthopaedic Surgery, Ninth People's Hospital, Shanghai Jiaotong University School of Medicine, Shanghai 200011, People's Republic of China; 3 Department of Orthopaedics, Changhai Hospital, the Second Military Medical University, Shanghai 200433, People's Republic of China

**Keywords:** Anatomy, Distal fibula, Distal tibia, Fracture

## Abstract

**Objective:**

This study aimed to investigate the feasibility of single incision for plating for the treatment of distal tibia and fibula fractures by a gross anatomic study.

**Methods:**

The anatomical structures of the anterolateral lower legs were identified. The lower leg length was measured from the top of fibular head to the tip of lateral malleolus. The distances between the extensor digitorum longus and anterior border of distal thirds of the tibia as well as the fibula were also measured. Additionally, their mutual relationships to the surrounding anatomical structures were described.

**Results:**

The distances from the proximal, middle, and distal thirds of the tibia to the extensor digitorum longus were 2.96 ± 0.46, 1.85 ± 0.25, and 2.15 ± 0.30 cm, respectively; the distances from the proximal, middle, and distal thirds of the fibula to the extensor digitorum longus were 1.82 ± 0.28, 2.09 ± 0.31, and 2.30 ± 0.27 cm, respectively. The results indicated that the safe gap from the distal tibia to extensor digitorum longus (EDL) was 1.6–3.4 cm and from the EDL to fibula was 1.5–2.6 cm. In addition, the average number of vascular pedicle in tibialis anterior, extensor hallucis longus, extensor digitorum longus, peroneus longus, and peroneus brevis was 2–3. Injuries generated by retracting medially and laterally in vascular pedicle could hardly affect the distal muscles.

**Conclusions:**

Therefore, we suggest that it is feasible to plate fractures of both the distal tibia and fibula through one anterolateral incision.

## Background

Fractures of the distal third of the tibia and fibula are relatively common fractures of long bones. The main etiologies of the fractures involve simple falls, motor vehicle trauma, or sports-related injuries as a result of axial compression and/or rotational forces [1-3]. Management of these fractures remains challenging to the surgeon. Traditional surgical methods include limited internal fixation with external fixation as well as open reduction and internal fixation. Open reduction and internal fixation is often favored for the improved ability to anatomically reduce displaced fractures, particularly articular fractures. However, open reduction and internal fixation generally involves two separate incisions: a medial incision to approach the distal tibia and a lateral incision to approach the distal fibula [2]. The double incisions may be associated with extensive soft tissue dissection and poor postoperative results, including soft tissue devitalization, skin sloughing, infection, and delayed union or non-union [4-7].

A single anterolateral incision technique has been advocated in several studies [7-9]. By using a single incision from the anterolateral side, the fibular fracture can be fixed and the lateral aspect of the distal tibia can be safely approached for internal fixation as described by Shantharam and co-workers, thus eliminating the need for two separate incisions [7]. Lateral approach for the distal tibia was used for 20 consecutive tibia fracture patients, and 17 patients achieved excellent or good subjective results [8]. Grose et al. have reported that a lateral approach for tibial pilon fractures is achieved in most fractures (93%), representing a good clinical efficacy [9]. However, there are few reports quantifying the feasibility of this single incision for plating for the treatment of distal tibia and fibula fractures by a gross anatomic analysis.

The objective of our study was to further describe the anatomic relationships between the distal tibia and fibula and the regional muscles, nerves, and blood vessels of the anterolateral compartment of the leg.

## Methods

For inclusion of cadavers in the study, the written informed consents were obtained from family members or legal guardians. In addition, all human studies were approved by the China Ethics Committee and performed in accordance with the ethical standards. This study was performed on 26 legs of 14 adult human embalmed cadavers (nine males and five females). Both legs were used in 12 cadavers, and a single leg was used in two cadavers (one right and one left). The mean age of human embalmed cadavers was 53 years (range, 42–71 years) at the time of death. All legs showed normal external appearances and no macroscopic evidence of previous trauma or degenerative changes. All measurements were made with the legs in the spontaneous extended positions and an average plantar flexion of 30°. All dissections were carried out as follows: the deep and superficial fascial layers were exposed, and then the anterior crural compartments were opened in the midline.

**Figure 1:**
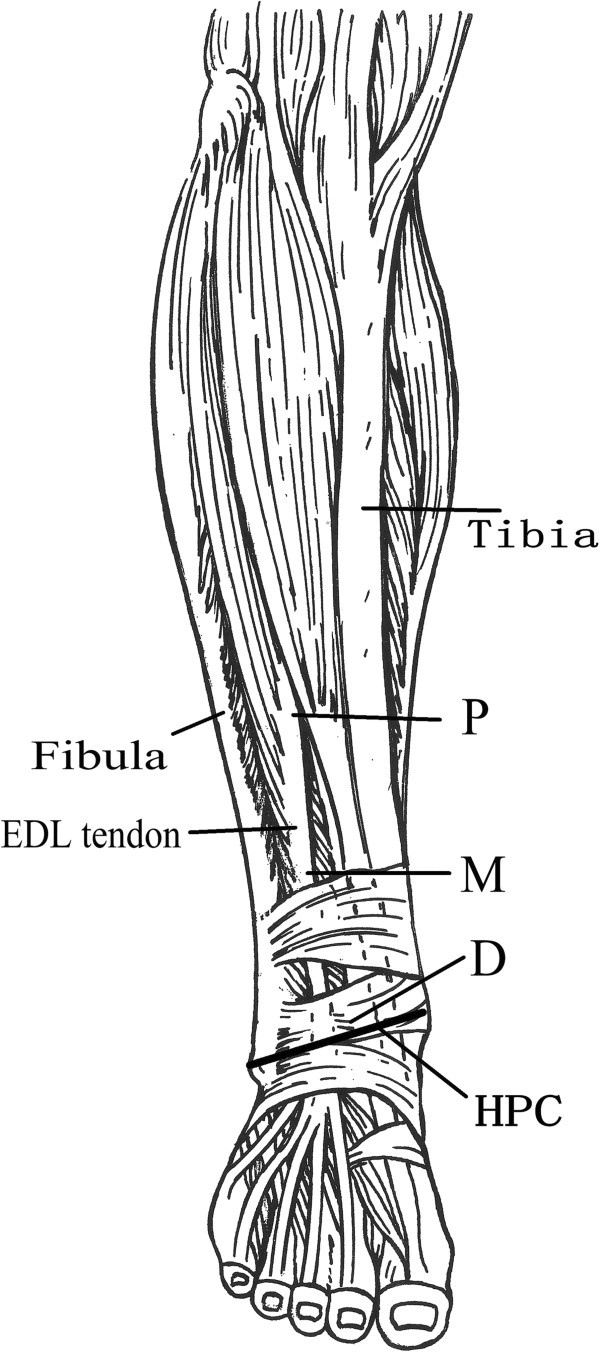
**Measurement diagram of the distances between the extensor digitorum longus and the tibia and fibula.***P* proximal measurement point of the distal third of the lower leg, *M* the middle measurement point, *D* distal measurement point, *EDL tendon* extensor digitorum longus tendon, *HPC* the highest point of the medial malleolus and the lateral malleolus of the connection.

After the anatomical structures of the anterolateral lower legs were identified, the lower leg length was measured from the top of fibular head to the tip of lateral malleolus. Measurements were conducted of the distances from the proximal, middle, and distal thirds of the tibia and fibula to the extensor digitorum longus (EDL) tendon, as shown in Figure 1. The distances between the EDL tendon and the anterior edge of tibia as well as the distances between the EDL tendon and the anterior edge of fibula were measured (accuracy value, 0.01 cm). Besides, distances between distal muscles (including tibialis anterior (TA), extensor hallucis longus (EHL), EDL, peroneus longus (PL), and peroneus brevis (PB)) and the highest point of medial malleolus and lateral malleolus of the connection (HPC) were measured (Figure 1). The number of vascular pedicle in distal muscles was observed. Each distance was measured three times.

Finally, the tibia was exposed by distracting the TA medially and the EHL, EDL, deep peroneal nerve, and anterior tibial vessels (DPN/ATV) laterally. To expose the distal third of the fibula, the EDL was retracted medially, and the peroneus longus and brevis were retracted laterally. The average values from measurements in all legs were calculated. Statistical analysis was carried out with SPSS for Windows 11.0 version.

## Results

### Distance guide

The lower leg lengths of each subject and the distances between the EDL tendon and the tibia and fibula of each specimen are shown in Table 1. The average length of 14 cadavers was 35.14 ± 2.10 cm. The average distances between the EDL tendon and the distal third of the tibia were 2.96 ± 0.46 cm (proximal), 1.85 ± 0.25 cm (middle), and 2.15 ± 0.30 cm (distal), respectively. The average distances between the EDL tendon and the distal third of the fibula were 1.82 ± 0.28 cm (proximal), 2.09 ± 0.31 cm (middle), and 2.30 ± 0.27 cm (distal), respectively (Table 1). The average distances between TA, EHL, EDL, PL, PB, and HPC were 11.3 ± 0.9, 5.0 ± 1.2, 6.6 ± 1.4, 5.3 ± 1.4, and 4.4 ± 1.4 cm, respectively (Table 2). The average number of vascular pedicle in TA, EHL, EDL, PL, and PB was 2.1 ± 0.7, 3.2 ± 0.7, 3.1 ± 0.7, 2.0 ± 0.7, 2.4 ± 0.6, respectively (Table 2).

**Table 1:** Distances between the extensor digitorum longus (EDL) tendon and the tibia and fibula, respectively

**Leg number**	**Leg length (cm)**	**Distance between EDL tendon and tibia (cm)**			**Distance between EDL tendon and fibula (cm)**		
		**Proximal**	**Middle**	**Distal**	**Proximal**	**Middle**	**Distal**
1	32.00	1.71	1.25	1.68	2.11	1.80	2.06
2	32.50	2.45	1.49	1.58	2.00	1.82	1.89
3	38.00	2.57	1.92	2.26	1.25	1.89	2.83
4	38.00	3.12	1.80	2.88	1.52	1.66	2.01
5	35.50	2.81	2.03	2.03	1.34	1.72	2.42
6	35.50	3.02	1.65	2.84	1.43	1.83	2.23
7	36.00	2.88	2.04	2.30	1.49	1.66	2.02
8	36.00	3.11	2.09	2.41	1.43	1.81	2.18
9	36.50	2.23	1.89	2.07	2.00	2.45	2.83
10	36.50	2.34	1.73	2.24	1.81	2.67	2.62
11	34.50	2.65	2.12	2.34	1.54	1.81	2.64
12	34.50	2.78	2.07	2.11	1.61	2.02	2.73
13	30.00	3.91	1.42	2.20	2.20	2.47	2.34
14	30.00	3.69	1.29	2.05	1.84	2.51	2.35
15	35.00	3.10	1.87	2.06	1.99	2.00	2.40
16	35.00	3.01	1.99	2.08	2.03	2.11	2.34
17	37.00	3.06	1.85	2.13	1.97	2.31	2.44
18	37.00	3.14	2.02	2.07	2.04	2.27	2.36
19	36.80	3.32	1.75	2.17	1.84	2.45	2.18
20	36.80	3.27	1.91	2.21	1.79	2.32	2.22
21	35.70	2.89	1.98	2.56	2.01	2.65	2.56
22	35.70	3.08	2.01	2.04	1.98	2.01	2.06
23	33.90	3.52	1.67	1.89	1.76	1.86	2.21
24	33.90	2.99	2.08	1.75	2.10	2.11	1.97
25	35.00	3.30	2.12	2.00	2.31	2.06	2.01
26	36.50	3.00	1.98	2.03	1.82	1.95	2.00
± *S*	35.14 ± 2.10	2.96 ± 0.46	1.85 ± 0.25	2.15 ± 0.30	1.82 ± 0.28	2.09 ± 0.31	2.30 ± 0.27

**Table 2:** Distances between distal muscles and HPC and the number of vascular pedicle in distal muscles

**Leg number**	**Distance between distal muscles and HPC (cm)**					**Number of vascular pedicle in distal muscles**				
	**TA**	**EHL**	**EDL**	**PL**	**PB**	**TA**	**EHL**	**EDL**	**PL**	**PB**
1	12.0	4.3	7.9	2.5	6.3	2	4	3	1	3
2	10.5	3.8	4.3	2.9	5.9	3	4	3	1	2
3	10.8	7.1	3.8	6.3	2.1	3	3	3	2	2
4	11.3	5.8	6.9	7.0	2.3	3	2	2	3	3
5	12.9	5.3	7.3	7.4	4.6	2	3	2	2	3
6	11.1	4.2	8.9	6.9	5.7	2	3	4	2	1
7	12.0	3.9	8.4	3.1	5.8	1	3	3	2	1
8	10.6	2.8	7.1	4.3	4.7	2	4	3	2	2
9	9.4	3.9	7.4	5.7	6.0	3	4	4	1	2
10	13.1	5.1	6.6	2.9	5.7	3	4	4	3	3
11	10.7	5.0	6.8	3.8	3.8	2	2	2	3	3
12	11.5	6.4	6.9	4.8	2.1	2	2	2	2	3
13	11.9	3.9	7.8	5.2	3.6	2	3	3	2	3
14	12.3	4.9	6.7	5.3	2.8	1	4	3	2	2
15	11.0	5.3	5.9	6.3	4.7	2	3	3	3	2
16	10.8	5.2	6.6	6.6	6.0	1	4	3	2	3
17	10.4	6.6	6.9	7.1	4.7	3	3	4	2	3
18	11.6	6.9	7.5	5.0	5.3	2	3	2	1	3
19	12.6	4.8	7.4	5.9	5.1	3	3	2	1	2
20	11.7	5.4	4.9	6.3	3.3	2	3	4	1	2
21	11.2	5.8	5.9	3.9	4.6	2	2	4	2	2
22	12.0	6.4	5.5	4.2	2.8	2	3	3	2	3
23	11.4	4.7	8.1	5.8	3.0	2	3	4	2	2
24	9.5	5.3	7.7	6.4	3.3	1	4	3	3	2
25	9.9	2.9	4.3	5.8	6.2	2	4	3	3	3
26	11.3	3.8	3.9	6.2	4.7	2	3	4	2	3
*S*	11.3 ± 0.9	5.0 ± 1.2	6.6 ± 1.4	5.3 ± 1.4	4.4 ± 1.4	2.1 ± 0.7	3.2 ± 0.7	3.1 ± 0.7	2.0 ± 0.7	2.4 ± 0.6

*TA* tibialis anterior, *EHL* extensor hallucis longus, *EDL* extensor digitorum longus, *PL* peroneus longus, *PB* peroneus brevis, *HPC* the highest point of the medial malleolus and the lateral malleolus of the connection.

### Anterior crural compartment dissection

**Figure 2:**
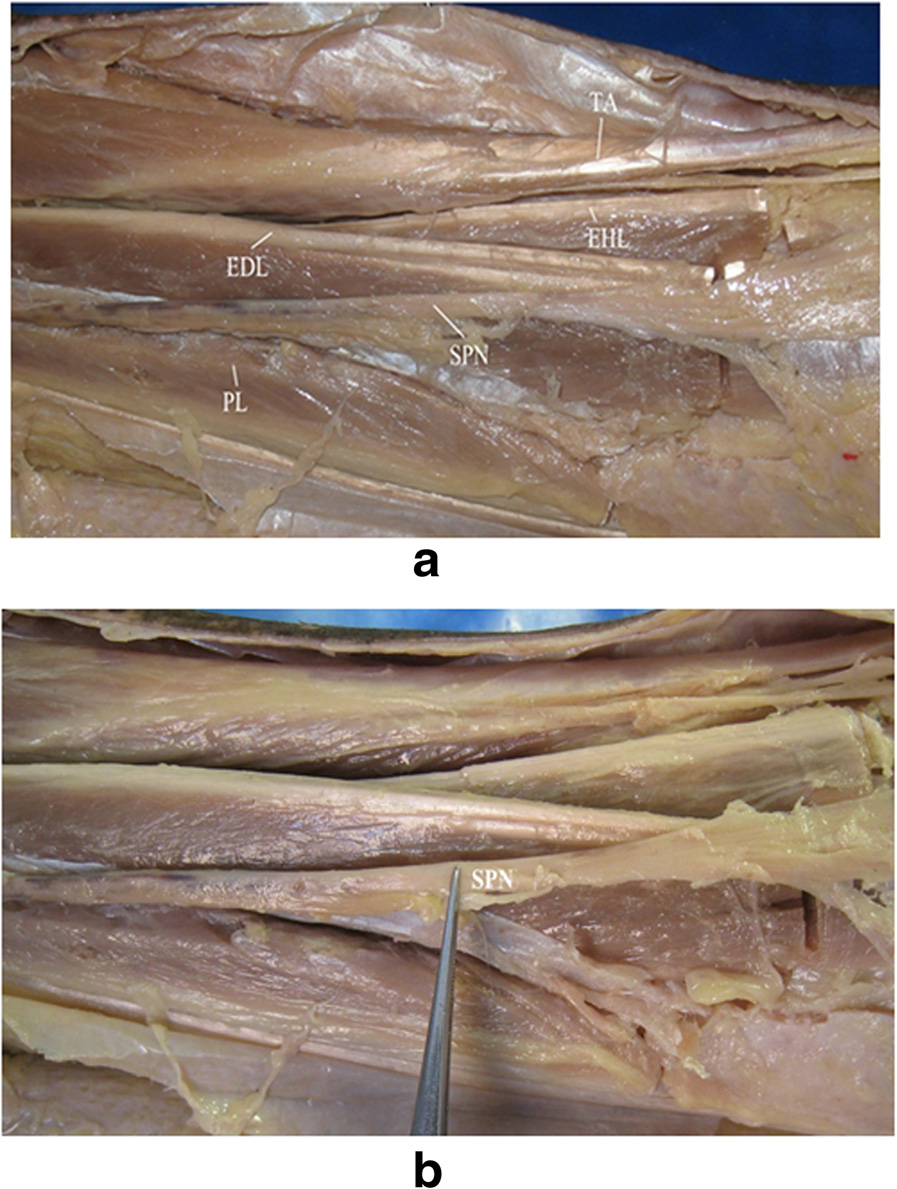
**The superficial structure and the location of SPN. (a)** In the superficial structure, each muscle of the anterior compartment was dissected, including tibialis anterior (*TA*), extensor hallucis longus (*EHL*), extensor digitorum longus (*EDL*), and the peroneus longus (*PL*) and brevis. **(b)** The location of superficial peroneal nerve (*SPN*).

**Figure 3:**
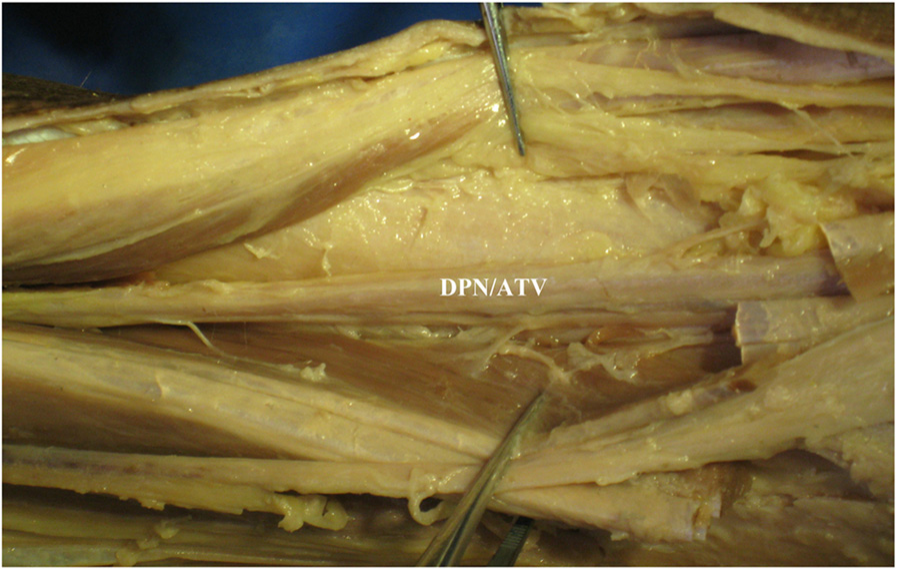
**Pedicle containing DPN/ATV.** In the deep structure, anterior to the interosseous membrane, the pedicle containing *DPN/ATV* rested upon the lateral surface of the distal tibia.

**Figure 4:**
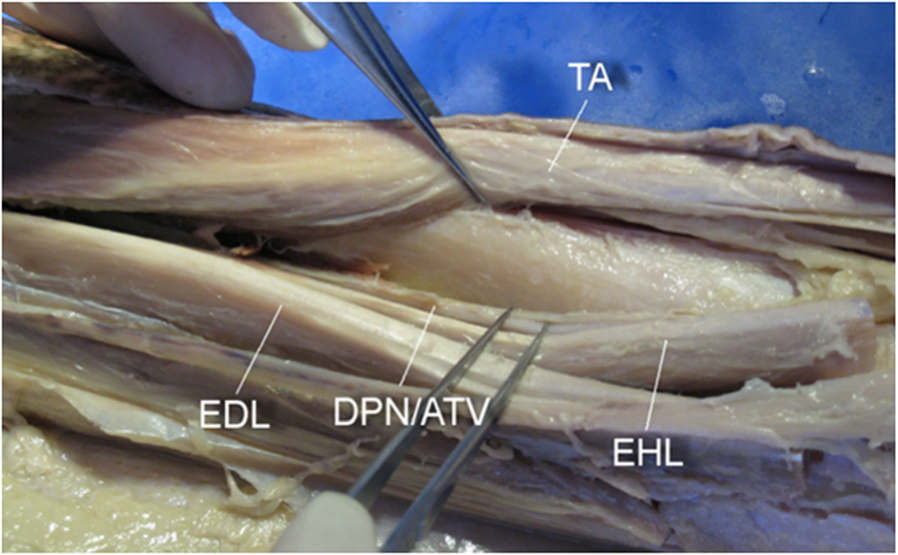
**Exposure of the tibia.** The tibia was successfully exposed by distracting the tibialis anterior (*TA*) medially and the extensor hallucis longus (*EHL*), extensor digitorum longus (*EDL*), deep peroneal nerve and the anterior tibial vessels (*DPN/ATV*) laterally.

An incision was made between the tibia and fibula of the anterolateral surface of the lower leg, which could be extended in both directions as required. In the superficial structure, each muscle of the anterior compartment was dissected, including TA, EHL, EDL, and the peroneus longus and brevis, as shown in Figure 2a. The superficial peroneal nerve (SPN) was observed between PL and PB, which passed over the junction of the lower third of the lower leg through the deep fascia to the subcutaneous tissue (Figure 2b). This structure was undamaged during dissection. In the deep structure, anterior to the interosseous membrane, the pedicle containing DPN/ATV rested upon the lateral surface of the distal tibia (Figure 3). When the distal tibia was exposed, the DPN/ATV could also be seen (Figures 3 and 4). These structures could not be damaged as long as enough care was paid.

### Exposure of tibia and distal third of the fibula

**Figure 5:**
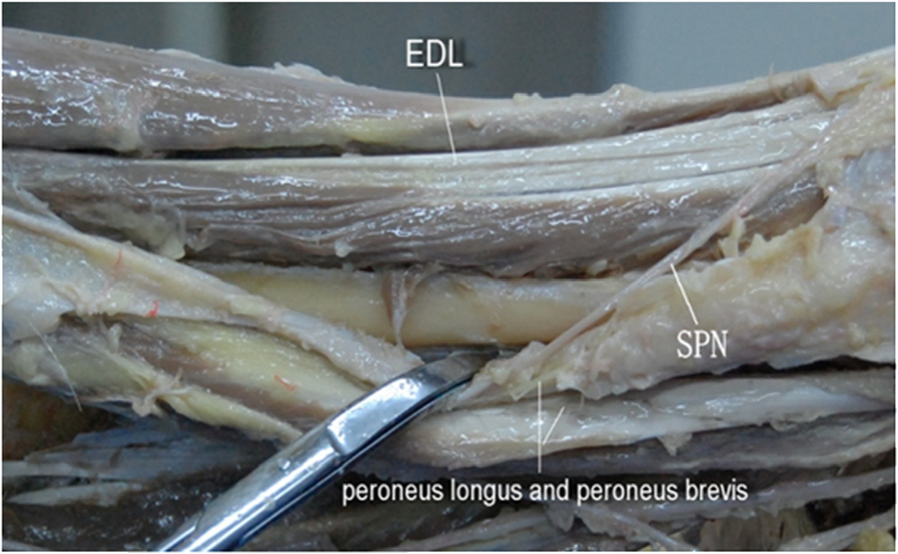
**Exposure of the distal third of the fibula.** The distal third of the fibula was successfully exposed by retracting extensor digitorum longus (*EDL*) medially and peroneus longus and brevis laterally. *SPN* superficial peroneal nerve.

The tibia was successfully exposed by distracting TA medially and EHL, EDL, DPN, and ATV laterally, as shown in Figure 4. The distal third of the fibula was also successfully exposed by retracting EDL medially and peroneus longus and brevis laterally, as shown in Figure 5.

## Discussion

The optimal treatment of unstable distal tibial and fibular fractures without articular involvement remains controversial. Traditional open reduction and internal fixation for the treatment of fractures of the distal third of the tibia and fibula is usually accomplished via the double-incision approach, which may contribute to severe soft tissue devitalization, skin sloughing, and infection complication [5-7,10]. A variety of treatment methods have been recommended to avoid these complications, including external fixation, intramedullary nailing, and percutaneous plate osteosynthesis [11,12]. However, each of these treatment options produces other challenges, such as extensive wound exposure, fracture propagation into the ankle, or nail failure due to inadequate hold, delayed union, and non-union [13-16].

Shantharam et al. proposed a single-incision treatment for the management of these fractures, and reported a good clinical efficacy [7]. The anatomical basis of an anterolateral approach to the distal tibia has also been subsequently described [17,18]. It is demonstrated that the SPN is always visualized in the subcutaneous tissues of the distal incision and is not at risk [18]. Understanding of the anatomy of the approach allows the development of improved operative techniques and outcomes. Our results further defined the anatomy of the distal tibia and fibula by a large series of cadaveric lower limbs.

The dissection results obtained in the present work demonstrated an obvious muscle gap and nerve interface in the anterolateral lower leg. Thus, an incision made in this region could avoid the major blood vessels and nerves, including the SPN between PL and PB in the superficial layers. In the deep structures, the course of the anterior tibial artery and vein and the deep peroneal nerve ran along the outer surface of the distal tibia. It was generally not easy to be damaged by retracting laterally for the protection under direct vision [17,18]. In addition, during the exposure of the tibia, the TA, EDL, and EHL muscles needed to be retracted medially and laterally to create a gap. According to the results described in Table 1, it was proposed that this gap could be retracted between 1.6 and 3.4 cm. While exposing the fibula, the gap between the EDL and the peroneus longus and brevis only needed to be retracted between 1.5 and 2.6 cm. The results indicated that the safe gap from the distal tibia to EDL was 1.6–3.4 cm and from the EDL to fibula was 1.5–2.6 cm. Moreover, the average number of vascular pedicle in TA, EHL, EDL, PL, and PB was 2–3. Thus, injuries generated by retracting medially and laterally in vascular pedicle could hardly affect the distal muscles.

There are several limitations in this study. The study may not capture all anatomic variations as a result of the small sample size. A larger sample size may result in narrower standard deviations. This study has been performed on cadaveric specimens, and thus, only the structural integrity of the saphenous and superficial peroneal nerves can be evaluated, not their functions. Additionally, measurements are performed on uninjured specimens. A distal tibia fracture or fibular shaft fracture may alter the course of adjacent neurovascular structures.

## Conclusions

In conclusion, we suggest that it is feasible to plate fractures of both the distal tibia and fibula through one anterolateral incision by a gross anatomic study. This operation can be performed through one anterolateral incision in a relatively simple manner. Our study confirms that single incision for plating for the treatment of distal tibia and fibula fractures is an efficient strategy to access the distal tibia and fibula.

## Competing interests

The authors declare that they have no competing interests.

## Authors’ contributions

HM designed this study and performed the statistical analysis. JZ carried out the study, together with BaY, and collected important background information. BiY drafted the manuscript. All authors read and approved the final manuscript.
